# Platycodon D protects human nasal epithelial cells from pyroptosis through the Nrf2/HO-1/ROS signaling cascade in chronic rhinosinusitis

**DOI:** 10.1186/s13020-024-00897-y

**Published:** 2024-03-04

**Authors:** Ruizhi Wang, Yongchun Wang, He Liu, Jinxiang Zhu, Caishan Fang, Weizhen Xu, Zesheng Lu, Yajie Yan, Weiping He, Yan Ruan, Min Zhou

**Affiliations:** 1 The First Clinical Medical School of Guangzhou University of Chinese Medicine, Guangzhou, 510405 China; 2 Department of Otolaryngology Head and Neck Surgery, The First Affiliated Hospital of Guangzhou University of Chinese Medicine, No.16 of Jichang Road, Baiyun District, Guangzhou, 510405 China; 3 Guangdong Clinical Research Academy of Chinese Medicine, Guangzhou, 510405 China; 4 Department of Allergy, The Third Affiliated Hospital of Sun Yat-Sen University, Guangzhou, 510630 China; 5 Hospital of Chengdu University of Traditional Chinese Medicine, Chengdu University of Traditional Chinese Medicine, Chengdu, 610000 China

**Keywords:** Pyroptosis, Chronic rhinosinusitis, Platycodon D, NLRP3 inflammasome, Nrf2/HO-1 antioxidant signaling pathway

## Abstract

**Background:**

Pyroptosis has been demonstrated being closely associated with the inflammatory progression in chronic rhinosinusitis (CRS). However, platycodon D (PLD) has emerged as a key anti-inflammatory mediator in the inflammatory progression of various respiratory diseases. This study aims at investigating whether PLD could reduce inflammatory progression of CRS by inhibiting pyroptosis.

**Methods:**

Nasal mucosal tissues from patients with CRS and the control group (simple nasal septal deviation) were analyzed for morphological difference using hematoxylin & eosin staining and for the expression of pyroptosis-related makers by immunofluorescence (IF). Human nasal epithelial cells (HNEpCs) were cultured and co-stimulated with lipopolysaccharide (LPS)/adenosine triphosphate (ATP) to construct an *in vitro* cellular model simulating CRS. After pretreatment with PLD, EthD-I staining, TUNEL staining, transmission electron microscopy (TEM), and GSDMD-NT detection were performed to evaluate pyroptosis markers. The NLRP3 inflammasome was detected by IF and western blotting (WB). Reactive oxygen species (ROS) were detected by H2DCFDA staining, and mitochondrial membrane potential was evaluated by JC-1 staining. Mitochondrial morphology and structure were observed using TEM. The Nrf2/HO-1 antioxidant signaling pathway was detected using WB.

**Results:**

The nasal mucosa structure of patients with CRS exhibited significant damage, with a marked increase in the expression of pyroptosis-related proteins compared with the control group. LPS/ATP co-stimulation resulted in an increased expression of IL-18 and IL-1β in HNEpCs, causing significant damage to nuclear and cell membranes, GSDMD-NT accumulation around the cell membrane, and intracellular NLRP3 inflammasome activation. Furthermore, it led to increased ROS expression, significantly decreased mitochondrial membrane potential, and damaged mitochondrial structure. However, pretreatment with PLD significantly reversed the aforementioned trends and activated the Nrf2/HO-1 antioxidant signaling pathway.

**Conclusions:**

The results of this study confirm that NLRP3-mediated pyroptosis plays a crucial role in the pathological process of nasal mucosal impairment in patients with CRS. PLD inhibits NLRP3-mediated pyroptosis, preventing inflammatory damage in HNEpCs of patients with CRS by activating the Nrf2/HO-1 antioxidant signaling pathway, which in turn reduces ROS production and ameliorates mitochondrial damage.

**Supplementary Information:**

The online version contains supplementary material available at 10.1186/s13020-024-00897-y.

## Introduction

Chronic rhinosinusitis (CRS) is a prevalent human respiratory disorder, with approximately 10% morbidity globally; as such, it poses a substantial burden on global heath [1]. As a chronic inflammatory disease of the sinus mucosa, CRS involves multiple pathophysiological mechanisms, including immunity, heredity, and microbial infection, with inflammatory injury and tissue remodeling of the nasal mucosa occurring throughout its pathogenesis [2]. Thus, inhibiting the inflammatory response represents an ideal approach to treat CRS [3]. However, the specific regulatory mechanism underlying CRS inflammation remains unclear, posing significant challenges for CRS management. Clarifying the specific inflammatory regulatory mechanism will be crucial in resolving this issue.

Pyroptosis, as a newly identified type of proinflammatory cell death, has been shown to participate in the pathophysiological process of various respiratory diseases, such as allergic rhinitis [4], chronic obstructive pulmonary disease, bronchial asthma, and bronchial lung cancer [5, 6]. However, the role of pyroptosis in CRS remains unclear. Pyroptosis is mediated by gasdermin D (GSDMD), which is activated by upstream inflammasomes in the cytoplasm [7]. Currently, the nucleotide-binding oligomerization domain-like receptor protein 3 (NLRP3) inflammasome is known to play a central role in regulating pyroptosis. When NLRP3 senses intracellular and extracellular risk signals, such as lipopolysaccharide (LPS), reactive oxygen species (ROS), adenosine triphosphate (ATP), and mitochondrial DNA [8], it can bind to apoptosis-associated speck-like protein containing CARD (ASC) via its PYD domain. Then, it recruits pro-cysteinyl aspartate-specific proteases-1 (pro-caspase-1) to assemble the inflammasome complex and generate cleaved Caspase-1 [9]. On the one hand, cleaved Caspase-1 cleaves downstream GSDMD, leading to the release of the GSDMD-N-terminal (GSDMD-NT) that induces the formation of holes in the cell membrane, ultimately triggering pyroptosis [10]. On the other hand, cleaved Caspase-1 can also cleave pro-interleukin-18 (pro-IL-18) and pro-IL-1β to generate mature IL-18 and IL-1β, respectively. These two molecules are released extracellularly through the holes formed by the GSDMD-NT in the cell membrane, inducing an inflammatory response in the surrounding tissues [11].

Numerous studies have confirmed that the overactivation of NLRP3 leads to uncontrolled inflammatory responses, tissue damage, and organ dysfunction [12]. Moreover, researchers have found high expression levels of NLRP3 in the nasal mucosal tissues of patients with CRS. Overexpression of NLRP3 and caspase-1 were observed in CRS with nasal polyps (CRSwNP), especially in eosinophilic nasal polyps [13]. It was also demonstrated that IL-17A and NLRP3 increased in nasal mucosa from patients with CRSwNP compared with those with CRSsNP [14]. This suggests that the NLRP3 inflammasome plays a crucial role in the inflammatory impairment of the nasal mucosa in CRS. Thus, it can potentially be targeted for the prevention and treatment of CRS by modulating the activation of the NLRP3 inflammasome and its mediated pyroptosis.

**Fig. 1:**
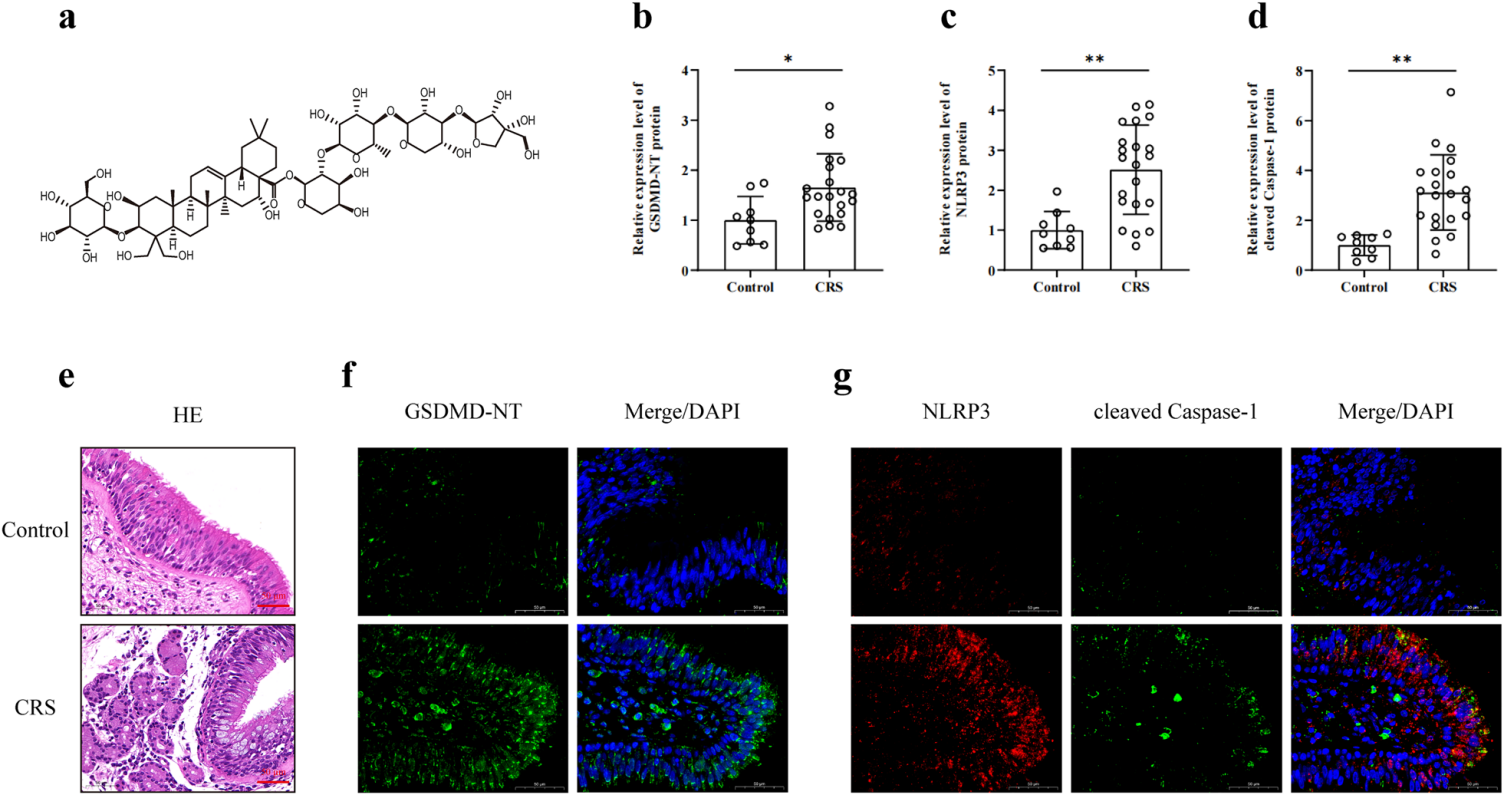
Chemical formula of Platycodon D and expression of pyroptosis-related markers in patients with CRS. **a** chemical formula of Platycodon D. **b**–**d** Statistical results of GSDMD, NLRP3, and cleaved Caspase-1. (Control group (n = 9), CRS group (n = 21); compared with Control group: *P < 0.05, **P < 0.01). **e** HE manifestation (Magnification = 400×, scale bar = 50 μm). **f**, **g** IF manifestation of GSDMD-NT, NLRP3, and cleaved Caspase-1 (Magnification = 630×, scale bar = 50 μm)

*Platycodon grandiflorus* (Jacq.) A.DC*.*, a natural medicinal plant commonly used in Southeast Asia, has been traditionally employed to prevent and treat various respiratory diseases, including acute lung injury [15] and pneumonia [16]. One of its main active components, platycodon D (PLD, the molecular structure of which is shown in Fig. 1a), has been confirmed to have a variety of pharmacological effects, including anti-inflammatory, antioxidant, anti-apoptosis, antiviral, antibacterial, and immune regulation effects, among which the anti-inflammatory and antioxidant effects are particularly prominent [17]. Wang et al. [18] demonstrated that PLD could inhibit the activation of the NLRP3 inflammasome in the intestinal tissue of mice by inhibiting the activation of the AMPK/NF-κB signaling pathway, ameliorating the injury of colon tissue. Similarly, Wu et al. [19] showed that PLD could regulate the activation of the NLRP3 inflammasome and effectively alleviate LPS-induced acute lung injury in rats. Choi et al. [20] showed that PLD significantly ameliorated the oxidative stress damage caused by H_2_O_2_ in mouse myoblast C2C12, mainly caused by the activation of the Nrf2/HO-1 signaling pathway, which reduced the generation of ROS. Zhang et al. [21] reported that PLD could also reduce neuroinflammatory reactions during the progression of Alzheimer's disease by activating the Nrf2/HO-1 signaling pathway. These studies indicate that PLD has promising anti-inflammatory and antioxidant effects and is closely related to the regulatory factors of pyroptosis. However, the effect of PLD on pyroptosis of human nasal mucosal epithelial cells (HNEpCs) in the pathological process of CRS remains poorly understood. Moreover, the specific regulatory mechanism by which PLD inhibits the activation of the NLRP3 inflammasome and exerts its antioxidant effect requires further investigation.

Therefore, in this study, nasal mucosal tissue samples from patients with CRS were collected to determine whether the NLRP3 inflammasome and pyroptosis are involved in the pathogenesis of CRS. Next, we established a cellular model *in vitro* by culturing HNEpCs originated from patients with CRS. LPS/ATP co-stimulation [22] was used to further explore whether PLD could regulate the activation of the NLRP3 inflammasome and pyroptosis via the Nrf2/HO-1 signaling pathway. This study aimed to provide a new direction for the clinical development of CRS-targeted therapeutic drugs.

## Materials and methods

### Patients and nasal mucosa specimens

Patients with simple nasal septal deviation and patients with CRS with nasal polyps (hereinafter referred to as CRS) admitted in the Otolaryngology department of the First Affiliated Hospital of Guangzhou University of Chinese Medicine from April 2022 to December 2023 were selected. Patients with CRS with nasal polyps were included according to the EPOS 2020 version of the CRS diagnostic criteria [1] (specific inclusion criteria and exclusion criteria are detailed in the Additional file 1). Nasal septum mucosa or inferior turbinate mucosa of patients with simple nasal septum deviation were collected as specimens of the control group. Uncinate process mucosa were collected as representative specimens of the CRS group. All patients gave written informed consent to participate in this study, and their clinical characteristics are shown in Additional file 3: Table S1. This study was approved by the Ethics Committee of the First Affiliated Hospital of Guangzhou University of Chinese Medicine (approval No. K-2022-031).

### Hematoxylin & eosin and immunofluorescence staining of tissue

Tissue specimens obtained from patients with CRS were immediately fixed in 4% paraformaldehyde and cut into coronal slices with a thickness of approximately 4 μm. The slices were stained with hematoxylin & eosin (HE) as previously described [23] and immunofluorescence (IF) was performed as previously described [14]. For IF, the slices were incubated with primary antibodies (Rabbit Anti-GSDMD-NT, AF4012, Affinity; Rabbit Anti-NLRP3, bs-10021R, Bioss; Rabbit Anti-cleaved Caspase-1, AF5418, Affinity; dilution: 1:200), then incubated with secondary antibodies (FITC labeled Goat Anti-rabbit IgG, ZF-0311, ZSGB-BIO; Alexa Fluor 594 labeled Goat Anti-rabbit IgG, ZF-0316, ZSGB-BIO; dilution: 1:50) at 24–26 ℃ in the dark for 60 min and finally stained with 4′,6-diamidino-2-phenylindole (DAPI, AR1176, Boster). The specimens were observed under a microscope (IX73, Olympus, Japan).

### Cell acquisition and modelling

The uncinate process mucosa of patients with CRS was collected for culturing primary HNEpCs as previously reported [24]. HNEpCs were cultured in PneumaCult^TM^-Ex Basal Medium (05008, STEMCELL) with 1% penicillin- streptomycin and incubated at 37 ℃ in a cell incubator (5% CO_2_). The experiment was conducted using 1–3 generations of cells. HNEpCs were randomly assigned to the following groups: control, model (LPS+ATP), PLD with different concentrations (0.625, 1.25, 2.5, 5.0, 10, 20, 40, and 80 μM), MCC950 (NLRP3 inhibitor), mito-TEMPO (Mitochondrial ROS scavenger), tert-butylhydroquinone (TBHQ, Nrf2 agonist), ML385 (Nrf2 inhibitor), and ML385+PLD groups.

After 48 h of incubation, the media in the control and model groups were replaced with PneumaCult^TM^-Ex Basal Medium. Media in the PLD groups were replaced with media containing the corresponding concentrations of PLD (122739-11-1, Chengdu Pufei De Biotech Co., Ltd.). Media in the MCC950, Mito-TEMPO, and TBHQ groups were replaced with media containing 5 μM MCC950 (256373-96-3, MedChemExpress), Mito-TEMPO (HY-112879, MedChemExpress), and TBHQ (HY-100489, MedChemExpress), respectively. Cells were then cultured continuously for 4 h and primed with 5 μg/mL LPS (L4391-1MG, Sigma) for 10 h. Thereafter, cells were stimulated with 5 mM ATP (A6419-1G, Sigma) for 2 h to activate NLRP3 inflammasome in the model, PLD, MCC950, Mito-TEMPO, and TBHQ groups.

Media in the ML385 and ML385+PLD groups were first replaced with media containing 5 μM ML385 (HY-100523, MedChemExpress). After cells were continuously cultured for 24 h, media in the ML385 and ML385+PLD groups were replaced with media containing 5 μM ML385 and 5 μM ML385+5 μM of PLD, respectively; the cells were then continuously cultured for 4 h and primed with 5 μg/mL LPS for 10 h. Thereafter, cells were stimulated with 5 mM ATP for 2 h to activate NLRP3 inflammasome in the ML385 and ML385+PLD groups.

### Cell viability assay

After treating HNEpCs as in section “Cell acquisition and modelling”, CCK-8 (CK04-500, DOJINDO) solution (1:10) was added to each well followed by incubation at 37 °C for 2 h. Then, the optical density (OD) value of each group was detected at 450 nm with a microplate reader (Multiskan GO, Thermo Scientific, USA). The cell survival rate of each group was calculated as follows: cell survival rate = (OD value of each treatment group − OD value of the background group)/(OD value of the control group − OD value of the background group) × 100%.

### LDH release determination

After treating HNEpCs as in section “Cell acquisition and modelling”, the cell culture supernatant was collected as the sample to be tested. The levels of LDH in the cell culture supernatant were measured using LDH kits (A020-2-1, Nanjing Jiancheng Bioengineering Institute) according to manufacturer instructions. LDH activity (U/L) in cell culture supernatant = (OD value of each sample to be tested − OD value of the control group)/(OD value of the standard − OD value of the blank) × 0.2 × 1000.

### Enzyme-linked immunosorbent assay (ELISA)

After treating HNEpCs as in section “Cell acquisition and modelling”, the cell culture supernatant was collected as the sample to be tested. The levels of IL-18 and IL-1β protein in the cell culture supernatant were measured using IL-18 ELISA kits (SEKH-0028, Solarbio) and IL-1β ELISA kits (SEKH-0002, Solarbio), respectively, according to manufacturer instructions.

### Cell membrane impairment

After treating HNEpCs as in section “Cell acquisition and modelling”, the cells were washed with PBS, and then dyed with Ethidium homodimer-I (EthD-I, HR8279, Beijing Baiaolaibo) solution (1:1000) for 5 min at 37 °C. Thereafter, cores were dyed with DAPI. The specimens were observed under a microscope. The proportion of EthD-I fluorescence-positive cells = (the number of cells with both red and blue fluorescence)/(the number of blue fluorescence cells) ×100%.

### Cell DNA impairment

After treating HNEpCs as in section “Cell acquisition and modelling”, the cells were washed with PBS and then fixed with 4% paraformaldehyde for 15 min. Thereafter, cells were incubated in PBS containing 0.5% Triton-X 100 for 30 min. Then, cellular DNA damage was measured using TUNEL kits (G1501, Servicebio) according to the manufacturer instructions. The specimens were observed under a microscope. The proportion of TUNEL fluorescence-positive cells = (the number of cells with both green and blue fluorescence)/(the number of blue fluorescence cells) ×100%.

### Ultrastructure observation of pyroptosis cell and mitochondria

After treating HNEpCs as in section “Cell acquisition and modelling”, cells were washed with PBS and fixed with 2.5% glutaraldehyde for 2 h at 4 °C. Samples were prepared for ultrastructure observation as previously reported [14]. The images were captured using a transmission electron microscope (HITACHI HT 7800, HITACHI, Japan).

### IF staining of cells

After treatment as in section “Cell acquisition and modelling”, HNEpCs were prepared for IF observation as previously described [14]. The specimens were stained with primary antibodies (Mouse Anti-GSDMD-NT, 66387-1-Ig, Proteintech; Rat Anti-NLRP3, MA5-23919, Invitrogen; dilution: 1:200) overnight at 4 °C. Subsequently, incubation with the fluorescent secondary antibody (FITC labeled Goat Anti-mouse IgG, ZF-0312, ZSGB-BIO; Alexa Fluor 594 labeled Goat Anti-rat IgG, ZF-0318, ZSGB-BIO; dilution: 1:50) was performed, followed by staining with DAPI at 37 °C. The specimens were observed under a confocal laser scanning microscope (Leica TCS SPE-II, Leica, Germany).

### Reactive oxygen species (ROS) detection

After treating HNEpCs as in section “Cell acquisition and modelling”, 20 µM H2DCFDA (HY-D0940, MedChemExpress) was added to HNEpCs followed by incubation at 37 °C in the absence of light for 30 min. The cells were washed with PBS and subsequently digested with 0.25% trypsin. Following centrifugation, the cells were resuspended in PBS and immediately analyzed using flow cytometry (BD LSRFortessaTM Cell Analyzer, BD Biosciences, USA).

### Mitochondrial membrane potential detection

After treating HNEpCs as in section “Cell acquisition and modelling”, 200 μM of JC-1 (HY-K0601, MedChemExpress) was directly added into the cellular medium so that the final concentration of JC-1 was 2 μM. The mixture was gently shaken and incubated at 37 °C for 20 min. After washing with PBS, red and green fluorescence was observed using a confocal laser scanning microscope. The ratio of red-to-green fluorescence was used to determine the mitochondrial membrane potential.

### Western blotting (WB)

Following treatment as in section “Cell acquisition and modelling”, the HNEpCs were digested with 0.25% trypsin and collected. Protein lysis buffer was added to each sample to extract the total cellular protein. Protein concentrations were determined using the BCA method and standardized to the same concentration (3 μg/μL). The total cell protein lysate was subjected to high-temperature boiling and denaturation. Subsequently, SDS-PAGE was performed, and the resulting blots were blocked with 5% skim milk powder at 37 °C for 1 h. Blots were then exposed to primary antibodies (Rabbit Anti-NLRP3, bs-10021R, Bioss; Mouse Anti-ASC, 67494-1-Ig, Proteintech; Rabbit Anti-cleaved Caspase-1, AF5418, Affinity; Rabbit Anti-GSDMD-NT, AF4012, Affinity; Rabbit Anti-IL-18, DF6252, Affinity; Mouse Anti-IL-1β, 66737-1-Ig, Proteintech; Rabbit Anti-Nrf2, 80593-1-RR, Proteintech; Mouse Anti-HO-1, 66743-1-Ig, Proteintech; Rabbit Anti-β-actin, GB11001, Servicebio; dilution: 1:1000) and incubated overnight at 4 °C. The following day, secondary antibodies (Horseradish Enzyme-labeled Goat Anti-Rabbit IgG, ZB-2301, ZSGB-BIO; Horseradish Enzyme-labeled Goat Anti-Mouse IgG, ZB-2305, ZSGB-BIO; dilution: 1:5000) were added and incubated at 24–26 °C for 1 h. An ECL developer was added, and a gel imager (ChemiDoc MP, Bio-Rad, USA) was used for imaging analysis. The integrated optical density (IOD) of each target band was measured using the Image-J software. The IOD ratios of target proteins and β-actin were used to quantify the expression levels of the target proteins.

### Statistical analysis

Image-J analysis software was used for analyzing the collected images. SPSS 24.0 software was used for statistical analysis, which was expressed as*‾x ± s*. All data were tested for normality and homogeneity of variance. One-way ANOVA was used for comparisons of the measurement data in each group. The LSD test was used for comparisons of homoscedasticity between groups, and Dunnett's T3 test was used for comparisons of homogeneity of variance between groups.* P* < 0.05 was considered statistically significant.

## Results

### Expression of pyroptosis-related markers increases in patients with CRS

HE staining (Fig. 1e) showed that epithelial tissues were arranged neatly and densely in the control group. Ciliated cells were predominant, in addition to a small number of goblet cells; a small number of glandular and inflammatory cells were observed beneath the epithelium. However, in the CRS group, the structure of the epithelia was disordered, goblet cells had proliferated, and substantial glandular hyperplasia and inflammatory cell infiltration were observed in subepithelial tissues.

IF detection (Fig. 1f, g) showed that, compared with the control group, the CRS group exhibited a significant increase in GSDMD-NT expression (*P* < 0.05), the key pyroptosis protein, in the epithelial layer of nasal mucosa. Furthermore, key components of NLRP3 inflammasome (NLRP3 and cleaved Caspase-1) that mediate the classic pyroptosis pathway were significantly increased in the nasal epithelial layer of patients with CRS (*P* < 0.01). This suggests the potential role of NLRP3-mediated pyroptosis in the inflammatory injury of CRS nasal mucosa.

### PLD improves cell damage and inhibits pyroptosis-related inflammatory factors

After network pharmacology analysis, 1483 CRS-related genes and 100 targets of PLD in the human body can be found (Additional file 4: Table S2). The R language was used to obtain 19 intersecting PLD and CRS-related genes (Additional file 2: Fig. S1a), which were imported into the STRING database to obtain a PPI network diagram (Additional file 2: Fig. S1b). According to the degree value of each node in the PPI network diagram, eight core targets were obtained using the MCODE software (Additional file 2: Fig. S1c). Analysis yielded 42 GO entries (*P* < 0.05; Additional file 2: Fig. S1d) and 26 KEGG pathways (*P* < 0.05; Additional file 2: Fig. S1e) using the DAVID database, most of which were closely related to inflammation. These results show that PLD may participate in and affect the inflammatory process of CRS, making it a potential drug for the treatment of this condition.

**Fig. 2:**
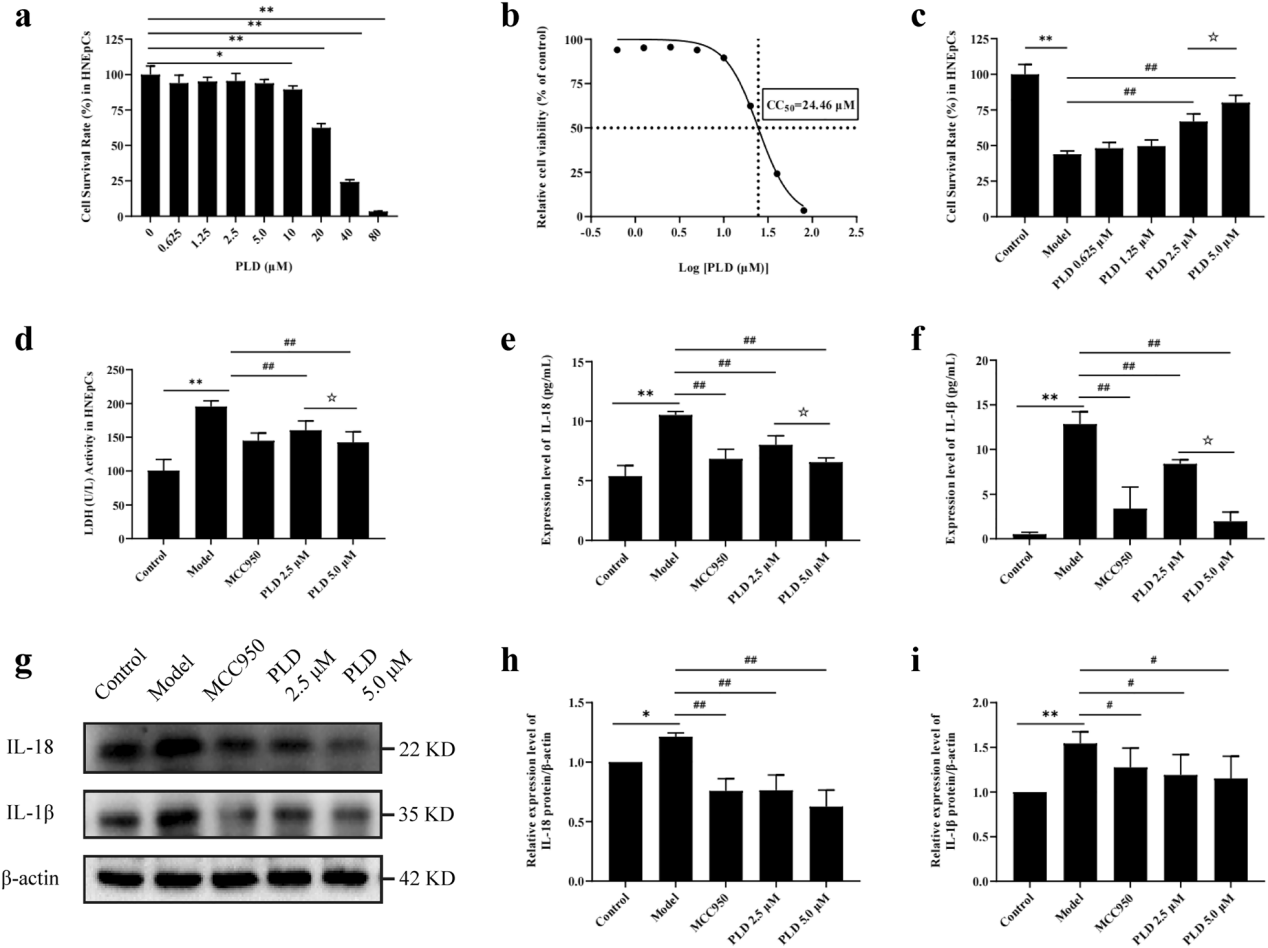
PLD improves cell damage and inhibited pyroptosis-related inflammatory factors. **a** Cell survival rate of HNEpCs after treatment with different concentrations of PLD without LPS/ATP co-stimulation. **b** The half inhibition rate of PLD on HNEpCs. **c**, **d** Cell survival rate and LDH release of HNEpCs after treatment with different concentrations of PLD and LPS/ATP co-stimulation. (n = 5). **e**, **f** Expression levels of IL-18 and IL-1β in cell supernatant (n = 5). **g**–**i** WB images and statistical results of IL-18 and IL-1β (n = 3) (compared with Control group: *P < 0.05, **P < 0.01; compared with Model group: #*P* < 0.05, ##*P* < 0.01; compared with PLD 2.5 μM group: ☆*P* < 0.05)

Next, we explored the role of PLD in CRS. First, we detected the cytotoxicity of PLD to HNEpCs. After 24 h of PLD pretreatment, PLD did not have a toxic effect on HNEpCs within the concentration range of 0–5.0 μM in CCK-8 test, while toxicity was observed at 10 μM (*P* < 0.05). The half inhibition rate of HNEpCs by PLD was estimated to be 24.46 μM (Fig. 2a, b).

Following LPS/ATP co-stimulation, the survival rate of HNEpCs decreased by approximately 50%, accompanied by an increase in LDH release (*P* < 0.01). However, pretreatment with PLD improved cell survival rate and inhibited LDH release in the range of 2.5–5.0 μM and exerted a protective effect on cells (*P* < 0.01). We found that 5.0 μM PLD was more effective than 2.5 μM PLD (*P* < 0.05). These results show that 5.0 μM PLD effectively attenuates the damage induced by LPS/ATP co-stimulation in HNEpCs (Fig. 2c, d).

WB (Fig. 2g, h, i) and ELISA (Fig. 2e, f) confirmed that PLD could inhibit both the expression of IL-18 (*P* < 0.01) and IL-1β (*P* < 0.05) in HNEpCs, and their extracellular release (*P* < 0.01). This effect was stronger in the 5.0 μM PLD group compared with that in the 2.5 μM PLD group, indicating that 5.0 μM PLD has a significant anti-inflammatory pharmacological effect.

### PLD inhibits pyroptosis

**Fig. 3:**
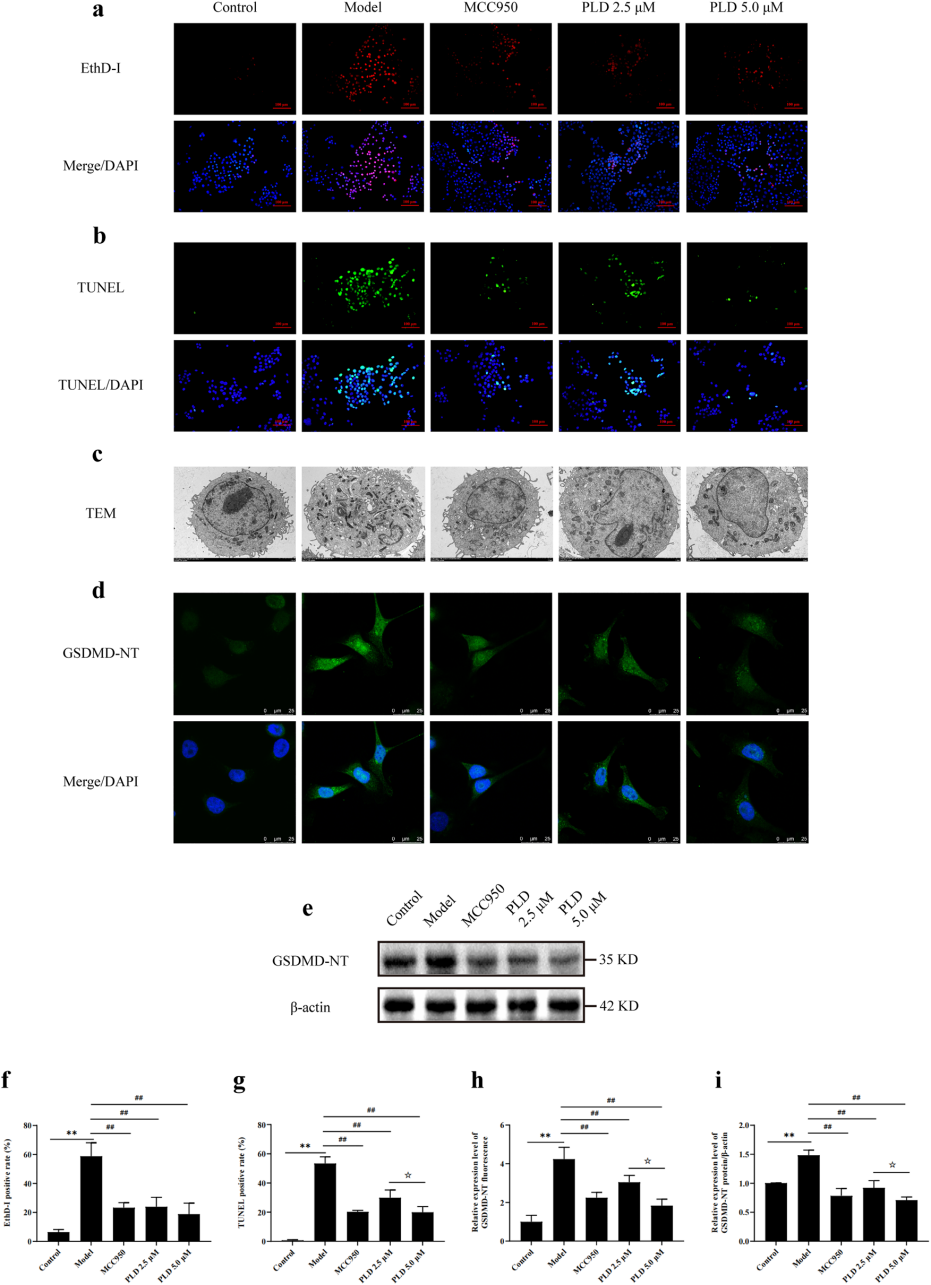
PLD suppresses the pyroptosis of HNEpCs. **a**, **f** Fluorescent images and statistical results of EthD-I (n = 5; magnification = 200×, scale bar = 100 μm). **b**, **g** Fluorescent images and statistical results of TUNEL (n = 5; magnification = 200×, scale bar = 100 μm). **c** TEM images of HNEpCs (magnification = 4000×, scale bar = 2 μm). **d**, **h** IF images and statistical results of GSDMD-NT (n = 5; magnification = 1000×, scale bar = 25 μm). **e**, **i** WB images and statistical results of GSDMD-NT (n = 3) (compared with Control group: **P < 0.01; compared with Model group: ##*P* < 0.01; compared with PLD 2.5 μM group: ☆*P* < 0.05)

The most important morphological feature of pyroptosis is the formation of pores in cell membranes. EthD-I is a nuclear fluorescent dye that can intercalate into the nuclear DNA double helix and emit red fluorescence when the integrity of the cell membrane is compromised. LPS/ATP co-stimulation significantly increased the number of EthD-I positive cells (*P* < 0.01), whereas pretreatment with PLD significantly reduced the number of EthD-I positive cells (*P* < 0.01). The declined tendency in the 5.0 μM PLD group was more obvious than that in the 2.5 μM PLD group, confirming that PLD attenuates cell membrane damage (Fig. 3a, f).

Pyroptosis is characterized by various morphological features, including DNA damage. TUNEL staining is a reliable method to detect nuclear DNA damage. Our results showed that the number of TUNEL positive cells increased significantly after LPS/ATP co-stimulation (*P* < 0.01), indicating that LPS/ATP co-stimulation led to nuclear DNA damage. However, treatment with PLD significantly reduced the number of TUNEL positive cells (*P* < 0.01), and 5.0 μM PLD was more effective than 2.5 μM PLD (*P* < 0.05), indicating that 5.0 μM PLD effectively inhibits nuclear DNA damage caused by LPS/ATP co-stimulation (Fig. 3b, g).

From TEM analysis, LPS/ATP co-stimulation caused structural impairment to the cell membrane and nucleus of HNEpCs, resulting in membrane breakage and porosity, cytoplasmic vacuolization, irregular nucleus shape, reduced size, and increased heterochromatin levels. Pretreatment with PLD significantly reduced the damage of HNEpCs (Fig. 3c).

GSDMD is the key factor that mediates the formation of cell membrane pores during pyroptosis. The GSDMD-NT released by GSDMD cleavage has pore-forming activity. Therefore, detection of GSDMD-NT is vital to determine pyroptosis. IF detection (Fig. 3d, h) showed that after LPS/ATP co-stimulation, GSDMD-NT fluorescent spots mainly clustered near the cell membrane (*P* < 0.01). However, pretreatment with PLD significantly inhibited the aggregation of GSDMD-NT fluorescent spots near the cell membrane (*P* < 0.01), indicating that PLD effectively inhibited GSDMD activation. WB (Fig. 3e, i) also showed that the expression of GSDMD-NT significantly increased after LPS/ATP co-stimulation (*P* < 0.01). However, pretreatment with PLD significantly inhibited the expression of GSDMD-NT (*P* < 0.01), and the concentration of 5.0 μM PLD was more effective than that of 2.5 μM PLD (*P* < 0.05). These findings demonstrate that 5.0 μM PLD has a strong pharmacological effect on inhibiting pyroptosis.

### PLD decreases NLRP3 inflammasome expression and activation

**Fig. 4:**
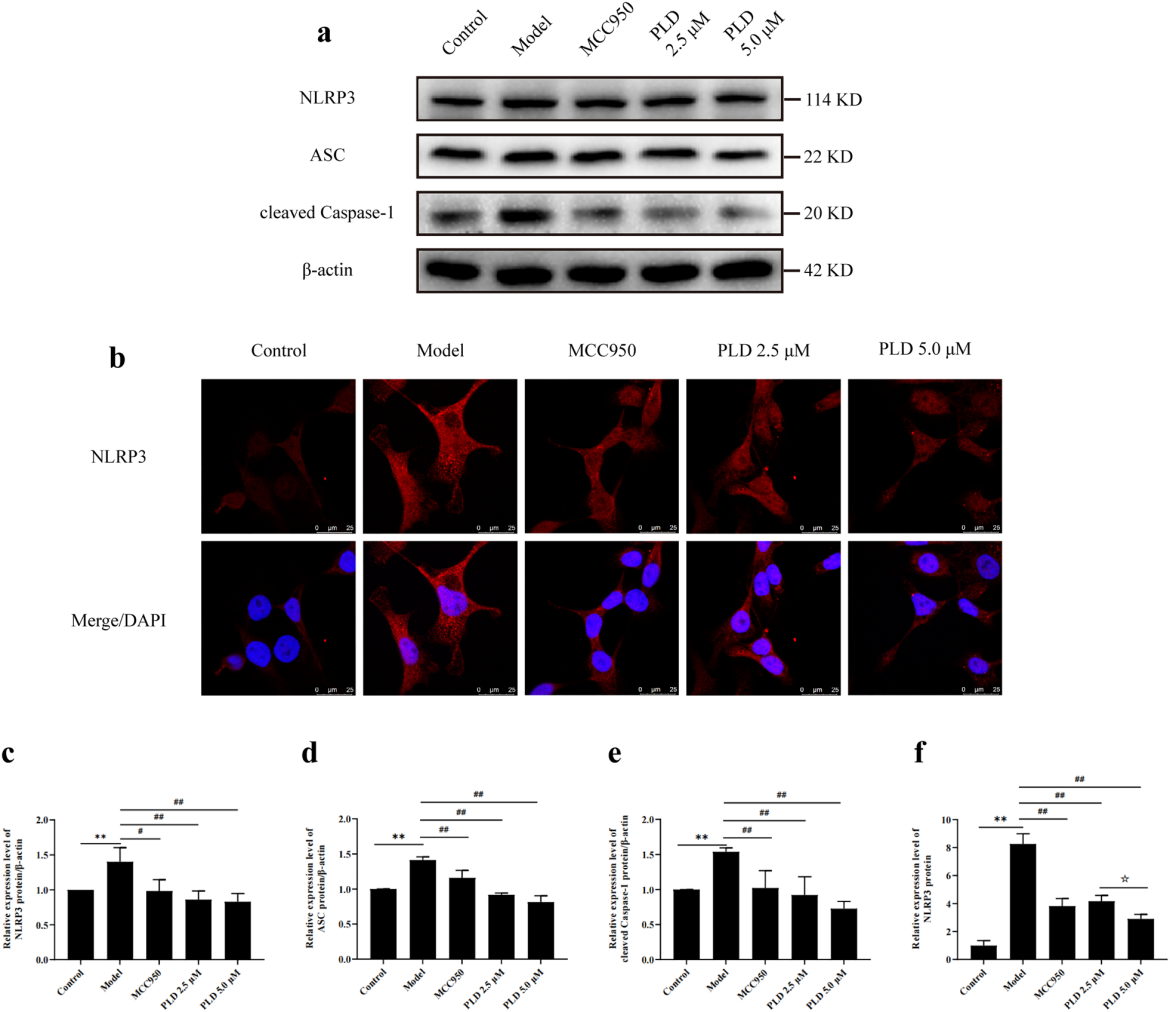
PLD inhibites the expression and activation of NLRP3 inflammasome. **a**, **c**–**e** WB images and statistical results of NLRP3, ASC, and cleaved Caspase-1 (n = 3). **b**, **f** IF images and statistical results of NLRP3 (n = 5; magnification = 1000×, scale bar = 25 μm) (compared with Control group: **P < 0.01; compared with Model group: #*P* < 0.05, ##*P* < 0.01; compared with PLD 2.5 μM group: ☆*P* < 0.05)

NLRP3 inflammasome is the key molecular complex that regulates the classical pyroptosis pathway. It consists of the initiating protein NLRP3, adaptor protein ASC, and effector protein Caspase-1. WB detection **(**Fig. 4a, c, d, e**)** demonstrated that LPS/ATP co-stimulation resulted in a significant increase in the expression levels of NLRP3, ASC, and cleaved Caspase-1 (*P* < 0.01). However, pretreatment with PLD significantly inhibited the expression of NLRP3, ASC, and cleaved Caspase-1 (*P* < 0.05). The decline tendency in the 5.0 μM PLD group was more obvious than that in the 2.5 μM PLD group. IF analysis (Fig. 4b, f) also confirmed that LPS/ATP co-stimulation led to a significant increase of NLRP3 fluorescent spots in cells (*P* < 0.01), whereas intervention with PLD significantly reduced the formation and expression of NLRP3 fluorescent spots (*P* < 0.01); 5.0 μM PLD was more effective than 2.5 μM PLD (*P* < 0.05). These findings show that 5.0 μM PLD effectively inhibits the assembly and activation of NLRP3 inflammasome.

### PLD reduces the generation of ROS and alleviates mitochondrial damage by regulating Nrf2 signaling molecules

**Fig. 5:**
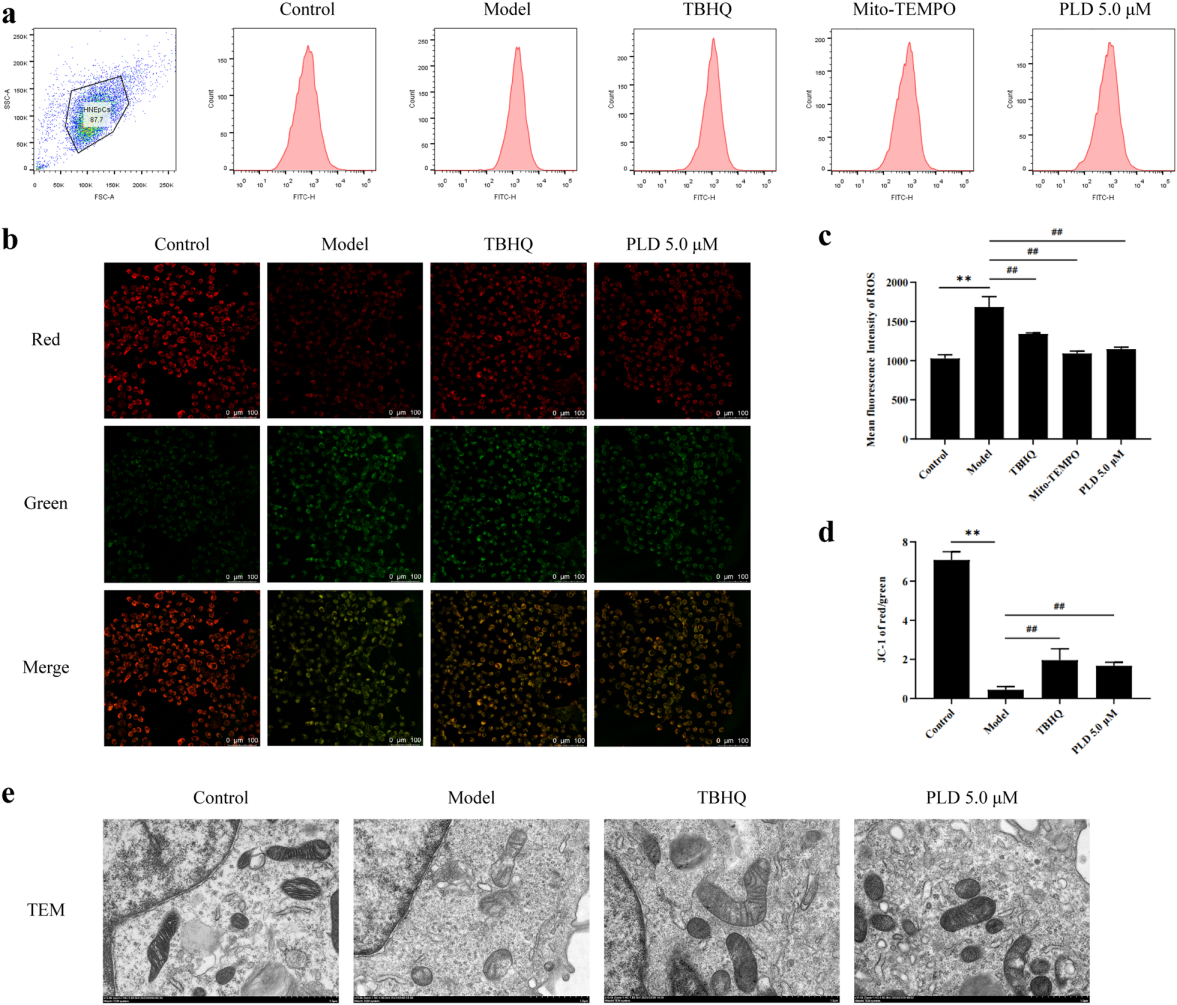
PLD reduces the generation of ROS and alleviates mitochondrial damage. **a**, **c** Flow cytometric analysis images and statistical results of ROS (n = 3). **b**, **d** Fluorescent images and statistical results of JC-1 red/green (n = 5; magnification = 200×, scale bar = 100 μm). **e** TEM images of mitochondria (magnification = 15000×, scale bar = 1 μm) (compared with Control group: **P < 0.01; compared with Model group: ##*P* < 0.01)

Activation of NLRP3 inflammasome is primarily regulated by oxidative stress-induced mitochondrial damage and ROS production. Flow cytometry analysis (Fig. 5a, c) showed that LPS/ATP co-stimulation significantly increased ROS expression (*P* < 0.01), whereas pretreatment with 5.0 μM PLD significantly reduced ROS production (*P* < 0.01). To confirm that ROS is mainly derived from mitochondria, we used Mito-TEMPO as a positive control. Mito-TEMPO significantly downregulated the expression of ROS in HNEpCs (*P* < 0.01), confirming that the intracellular ROS production after LPS/ATP co-stimulation mainly originated from the mitochondria.

To confirm the protective effect of PLD on mitochondria, we determined the mitochondrial membrane potential (Fig. 5b, d), and observed mitochondrial morphology (Fig. 5e). After LPS/ATP co-stimulation, the ratio of red/green fluorescence of JC-1 decreased significantly (*P* < 0.01), indicating serious mitochondrial membrane potential loss and mitochondrial impairment. However, treatment with 5.0 μM PLD increased the red/green fluorescence ratio of JC-1 (*P* < 0.01), suggesting that PLD alleviated mitochondrial damage. The TEM results showed that intracellular mitochondria significantly swelled after LPS/ATP co-stimulation, while PLD treatment could help improve this situation.

**Fig. 6:**
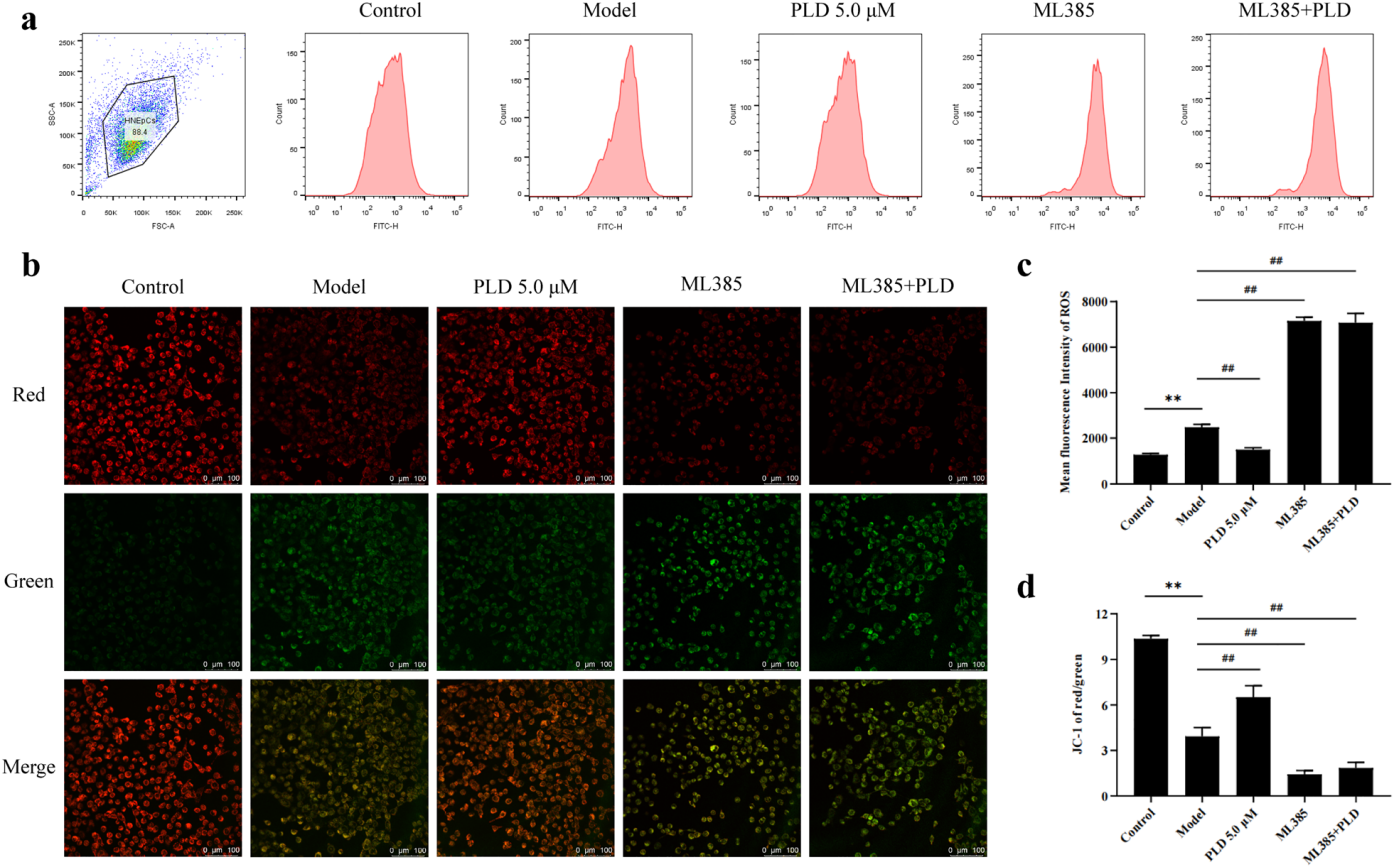
PLD reduces the generation of ROS and alleviates mitochondrial damage by regulating Nrf2 signaling molecules. **a**, **c** Flow cytometry and statistical analysis results of ROS expression (n = 3). **b**, **d** Mitochondrial membrane potential expression and statistical analysis results (n = 5; magnification = 200×, scale bar = 100 μm) (Compared with Control group: **P < 0.01; Compared with Model group: ##*P* < 0.01)

Flow cytometry (Fig. 6a, c) showed that when HNEpCs were pre-treated with ML385, the inhibitor of Nrf2, the expression of ROS was further increased compared with that in the model group (*P* < 0.01). In addition, the effect of PLD on ROS expression was also significantly inhibited (*P* < 0.01). The confocal laser experiment (Fig. 6b, d) showed that after pre-treatment with ML385, the level of mitochondrial membrane potential in HNEpCs was further decreased compared with that in the model group (*P* < 0.01). In addition, the ability of PLD to recover mitochondrial membrane potential was also significantly inhibited (*P* < 0.01).

### PLD inhibits NLRP3-mediated pyroptosis by activating the Nrf2/HO-1 antioxidant signaling pathway

**Fig. 7:**
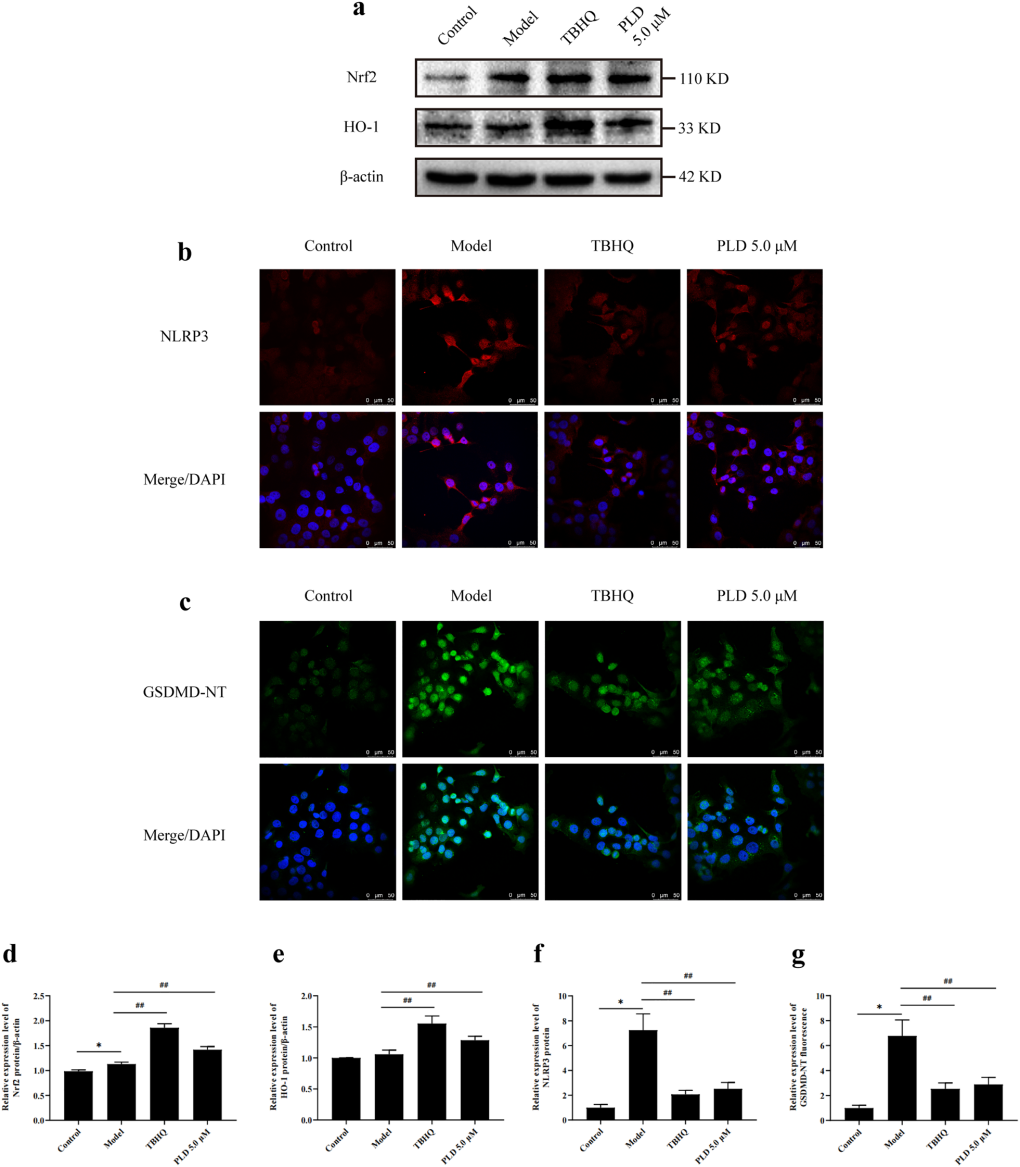
PLD activates the Nrf2/HO-1 antioxidant signaling pathway. **a**, **d**, **e** WB images and statistical results of Nrf2 and HO-1 (n = 3). **b**, **c**, **f**, **g** IF images and statistical results of NLRP3 and GSDMD (n = 5; magnification = 400×, scale bar = 50 μm) (compared with Control group: *P < 0.05, **P < 0.01; compared with Model group: ##*P* < 0.01)

The Nrf2/HO-1 signaling pathway is an important endogenous antioxidant stress pathway. WB data (Fig. 7a, d, e) showed that 5.0 μM PLD significantly upregulated the expression of Nrf2 and HO-1 in HNEpCs (*P* < 0.01), indicating that PLD activated the Nrf2/HO-1 signaling pathway. IF detection (Fig. 7b, c, f, g) showed that compared with PLD treatment, pretreatment with an Nrf2 agonist, TBHQ, also could inhibit the expression of NLRP3 and GSDMD in HNEpCs (*P* < 0.01). This suggests that the pharmacological effect of PLD on NLRP3-mediated pyroptosis is related to activation of the Nrf2/HO-1 antioxidant signaling pathway.

**Fig. 8:**
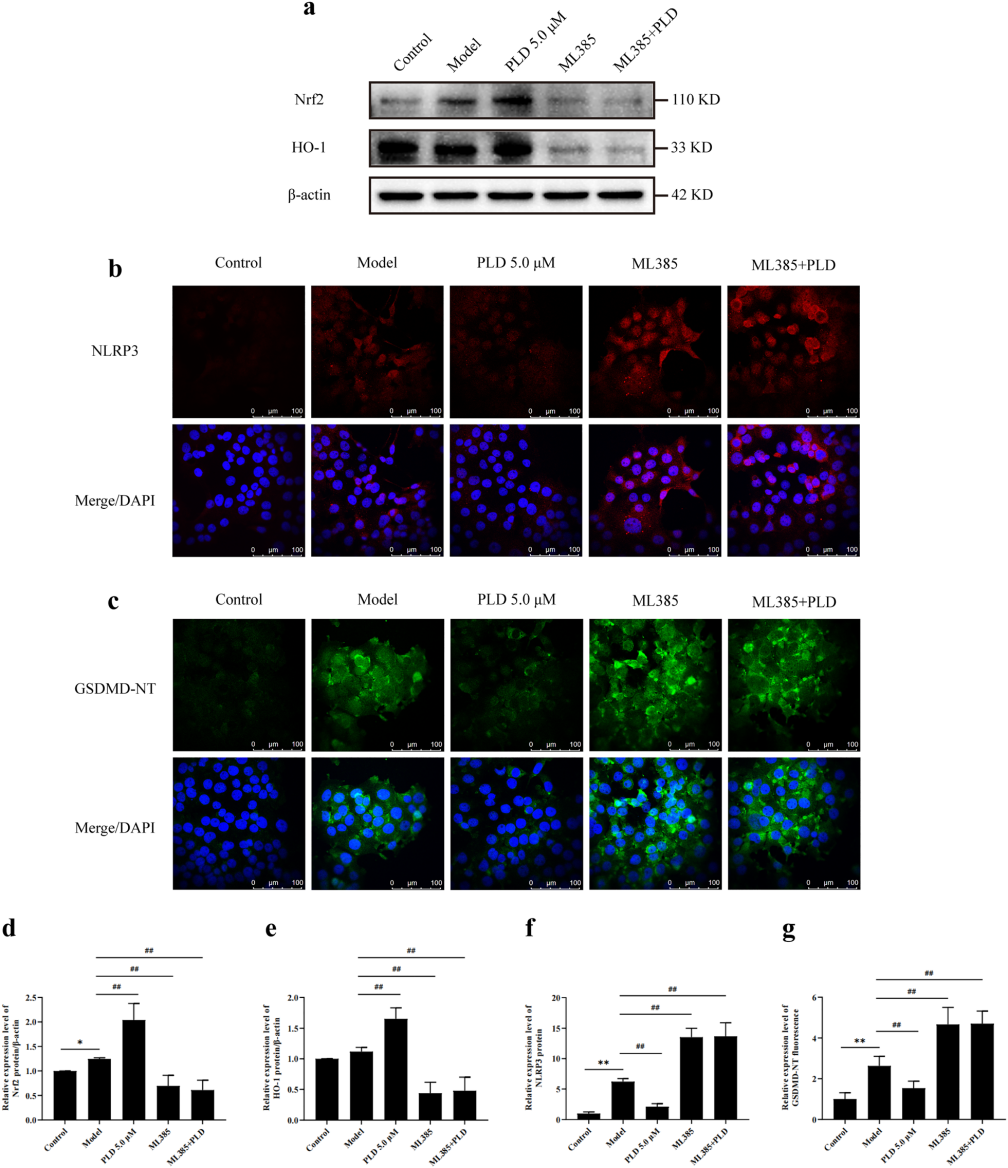
PLD inhibites NLRP3-mediated pyroptosis by activating the Nrf2/HO-1 antioxidant signaling pathway. **a**, **d**, **e** WB images and statistical results of Nrf2 and HO-1 (n = 3). **b**, **c**, **f**, **g** IF images and statistical results of NLRP3 and GSDMD (n = 5; magnification = 400×, scale bar = 100 μm) (compared with Control group: *P < 0.05, **P < 0.01; compared with Model group: ##*P* < 0.01)

WB (Fig. 8a, d, e) showed that after pre-treatment with ML385, the expression of Nrf2 and HO-1 in HNEpCs were significantly decreased compared with that in the model group (*P* < 0.01,). In addition, the ability of PLD to upregulating the expression of Nrf2 and HO-1 was also significantly inhibited (*P* < 0.01). IF analysis (Fig. 8b, c, f, g) showed that after pre-treatment with ML385, the expression levels of NLRP3 and GSDMD in HNEpCs were further increased compared with that in the model group (*P* < 0.01). In addition, after HNEpCs were pre-treated with ML385, the ability of PLD to inhibit the expression of NLRP3 and GSDMD was also significantly inhibited (*P* < 0.01).

## Discussion

CRS is characterized by inflammation of the nasal cavity and paranasal sinuses, with high expression of various inflammatory mediators in nasal mucosal tissues, which is associated with a poor prognosis [25, 26]. The inflammatory mediators can recruit different inflammatory cells, such as neutrophils [27], macrophages [28], mast cells [29], and eosinophils [30], to nasal mucosal tissue, further contributing to the exacerbation of the local inflammatory response. Our previous study also demonstrated that the expression of IL-18 and IL-1β was significantly increased in the nasal mucosa of patients with CRS [31]. This indicates that the chronic inflammatory damage of nasal mucosa is the key pathological feature of CRS. Therefore, targeting the excessive inflammatory response and improving damage to nasal mucosa are crucial for treating CRS [1]. However, owing to the unclear molecular mechanisms underlying the mucosal inflammatory response in CRS, CRS therapy remains a challenge. Therefore, there is an urgent need to further clarify the inflammatory mechanism underlying nasal mucosal injury in CRS.

Pyroptosis is a programmed cell death process mediated by GSDMD and closely related to inflammatory responses [7]. Currently, pyroptosis has been implicated in the pathophysiology of various inflammatory diseases, such as ischemia-reperfusion injury [32], cirrhosis [33], and Alzheimer’s disease [34]. Morphologically, pyroptotic cells exhibit condensed nuclei, chromatin DNA breaks, and positive TUNEL staining, similar to apoptotic cells [35]. However, more importantly, pyroptosis is characterized by GSDMD-NT, which can lead to the formation of molecular pores with diameters of 10–16 nm on the cell membrane, ultimately resulting in the destruction of the cell membrane [36]. The formation of these pores leads to increased intracellular osmolarity and cellular edema [37]. Eventually, the cell membrane ruptures, releasing inflammatory mediators, such as IL-18 and IL-1β, which induces an inflammatory response [38]. In the context of CRS, GSDMD-NT was found to be highly expressed in the nasal mucosa (Fig. 1), suggesting that pyroptosis plays a nonnegligible role in the pathophysiology of CRS. Therefore, inhibiting pyroptosis to reduce inflammation may be a promising strategy for treating CRS.

PLD, a naturally active component extracted from *Platycodon grandiflorus*, has been reported to have potent anti-inflammatory effects. However, its potential use for treating CRS and its regulatory effects on pyroptosis have not been thoroughly investigated. The findings of network pharmacology analysis demonstrated the therapeutic potential of PLD in the treatment of CRS (Additional file 2: Fig. S1 and Additional file 4: Table S2). In our study, an HNEpC inflammatory injury model was established to mimic the inflammatory injury state of HNEpCs in CRS by using LPS/ATP co-stimulation. LPS/ATP co-stimulation resulted in significant cell membrane and nuclear damage, upregulation of GSDMD-NT protein expression, and aggregation of GSDMD-NT fluorescent specks around the cell membrane, indicating pyroptosis induction in HNEpCs (Fig. 3). Moreover, LPS/ATP co-stimulation led to a significant increase in IL-18 and IL-1β expression in intracellular and cellular supernatants, accompanied by increased LDH release and decreased cell survival rate (Fig. 2), which indicated the involvement of pyroptosis in the inflammatory injury process of HNEpCs. However, PLD could significantly improve cell membrane and nuclear damage. PLD could also downregulate GSDMD-NT expression and inhibit GSDMD-NT fluorescent speck aggregation around the cell membrane (Fig. 3), indicating its inhibitory effect on pyroptosis of HNEpCs. Meanwhile, PLD could downregulate IL-18 and IL-1β expression in intracellular and cellular supernatants, reduce LDH release, and increase cell survival rate, suggesting that PLD has the potential to inhibit pyroptosis and alleviate inflammatory damage to HNEpCs.

As an important pattern recognition receptor, NLRP3 inflammasome plays a central role in regulating pyroptosis. It consists of the initiating protein NLRP3, adaptor protein ASC, and effector protein Caspase-1. When activated by endogenous or exogenous danger signals, NLRP3 indirectly activates Caspase-1 via ASC and targets GSDMD, which directly mediates pyroptosis and the release of inflammatory mediators [9, 10]. NLRP3 and Caspase-1 are upregulated in the nasal mucosa of patients with CRS. Inhibiting NLRP3 activity with MCC950 and silencing NLRP3 expression with siRNA can prevent pyroptosis and reduce inflammation in HNEpCs [14]. This suggests that blocking the activation of NLRP3 inflammasome may be an optional way to inhibit pyroptosis in CRS. In this study, we also found increased expression of NLRP3 and cleaved Caspase-1 in the nasal mucosa of patients with CRS (Fig. 1), which further verified the importance of NLRP3 in CRS. *In vitro* experiments showed that LPS/ATP co-stimulation significantly upregulated the expression of NLRP3, ASC, and cleaved Caspase-1 in HNEpCs. However, PLD significantly reduced the expression of NLRP3, ASC, and cleaved Caspase-1 after LPS/ATP co-stimulation (Fig. 4), inhibiting cell pyroptosis and cell damage. This suggests that PLD exerted a protective effect on HNEpCs by inhibiting NLRP3 inflammasome assembly and activation.

NLRP3 activation is a highly intricate process involving three pathways [39]: high ATP concentration in the extracellular region leading to a large outflow of intracellular potassium ions, lysosomal rupture, and oxidative stress (i.e., ROS production). Recent studies have shown that oxidative stress-induced mitochondrial damage and ROS production are key regulatory signals for activating the NLRP3 inflammasome [40, 41]. Our study showed that LPS/ATP co-stimulation caused a significant reduction in the mitochondrial membrane potential of HNEpCs (Fig. 5). It also resulted in evident structural damage, such as swelling, accompanied by a significant increase in intracellular ROS production. Moreover, our study also showed that PLD and TBHQ (Nrf2 agonist) significantly improved mitochondrial membrane potential decline and mitochondrial structural damage while reducing ROS generation (Fig. 5). The results were the opposite after pre-treatment with ML385, the inhibitor of Nrf2 (Fig. 6). This suggests that PLD reduces the generation of ROS and alleviates mitochondrial damage by regulating Nrf2 signaling molecules.

Nrf2 is a member of the cap ‘n’ collar (CNC) transcription factor family that regulates the expression of downstream antioxidant genes and is an important part of the Nrf2/ARE endogenous antioxidant stress pathway [42, 43]. When the body is exposed to oxygen free radicals or endogenous toxins, Nrf2 uncouples, enters the nucleus, and binds to ARE to upregulate the expression of downstream antioxidant genes [44]. Wang et al. [45] found that a small-molecule Nrf2 agonist could significantly inhibit NLRP3 inflammasome activation in macrophages, which was attributed to Nrf2 blocking NLRP3 initiation, preventing Caspase-1 cleavage, and reducing IL-1β secretion. However, Bian et al. [46] found that when Nrf2 expression was reduced in depressed mice, the expression of NLRP3, Caspase-1, and IL-1β increased significantly, indicating that Nrf2 can exert anti-inflammatory effects by regulating NLRP3 activation. HO-1, downstream of the Nrf2/ARE signaling pathway, is a potential endogenous cytoprotective enzyme with promising antioxidant and anti-inflammatory activities. Luo et al. [47] observed that heme, a potent inducer of HO-1, inhibited the expression of NLRP3, ASC, and Caspase-1 in lung tissue and reduced the secretion of IL-1β and IL-18 by reducing the production of ROS in sepsis. Similarly, Kim et al. [48] reported that HO-1 overexpression inhibited NLRP3 inflammasome activation, confirming the regulatory relationship between HO-1 and NLRP3. These studies indicate that the upregulation of HO-1 not only maintains the balance of ROS production, but also counteracts the inflammatory response in various tissues.

In this study, LPS/ATP co-stimulation slightly upregulated the expression of Nrf2, indicating that the antioxidant capacity of HNEpCs was mobilized by ROS. However, after PLD and TBHQ treatment, the expression of Nrf2 and HO-1 was significantly upregulated (Fig. 7), indicating that PLD could further activate the Nrf2/HO-1 antioxidant signaling pathway in HNEpCs. Additionally, ML385 inhibited the regulation of PLD on NLRP3 and GSDMD-NT after LPS/ATP stimulation (Fig. 8). These findings further support that the pharmacological effect of PLD on NLRP3-mediated pyroptosis is related to the activation of the Nrf2/HO-1 antioxidant signaling pathway.

Nevertheless, there are a few limitations to this study. First, our investigation provided only *in vitro* clarification of the pharmacological effect and molecular mechanism of the inhibition of HNEpC pyroptosis by PLD. Further animal experiments are needed to lay a firmer theoretical foundation for the clinical application of PLD in the treatment of CRS. Moreover, further discussion is needed as to whether PLD could interfere with NLRP3 activation by inhibiting potassium outflow and lysosomal rupture.

## Conclusion

**Fig. 9:**
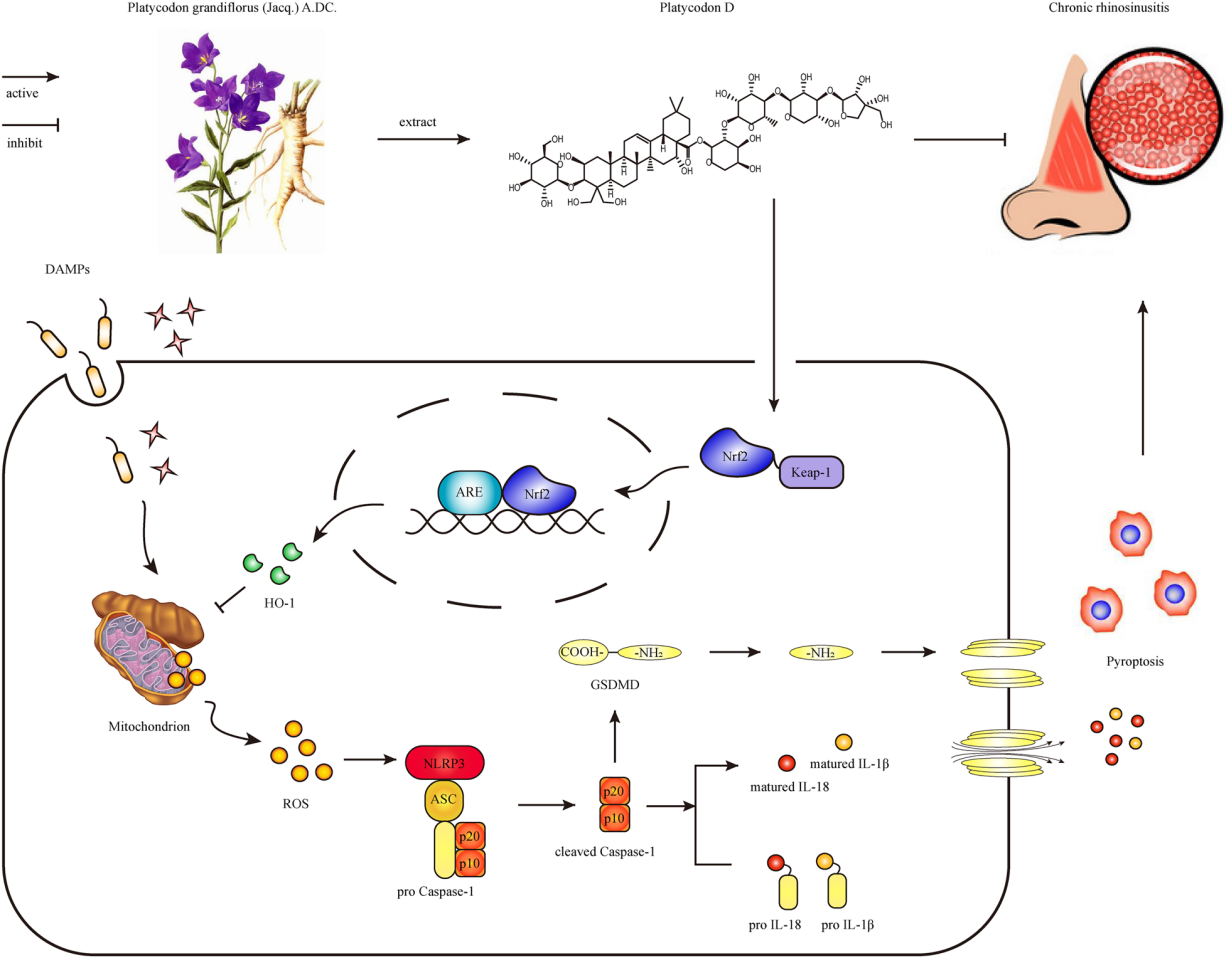
PLD improves mitochondrial damage by activating the Nrf2/HO-1 antioxidant signaling pathway, reducing ROS production, and inhibiting the activation of NLRP3 inflammasome, thereby inhibiting pyroptosis and the inflammatory damage in HNEpCs of patients with CRS

Our study confirms that pyroptosis, mediated by NLRP3 inflammasome, is involved in the inflammatory impairment process of nasal mucosa in patients with CRS. Moreover, this research demonstrates that PLD may inhibit NLRP3-mediated pyroptosis through the Nrf2/HO-1 signaling pathway in CRS. PLD plays an essential role in reducing ROS production and inhibiting the activation of NLRP3 inflammasome, thereby inhibiting pyroptosis and inflammatory damage in the HNEpCs of patients with CRS, as shown in Fig. 9. These findings provide a new research direction and a reference for the clinical development of targeted CRS therapy drugs.

### Supplementary Information

**Additional file 1.** The specific inclusion criteria and exclusion criteria of patients and the method of network pharmacology.

**Additional file 2:** **Fig S1.** Network pharmacology confirmed that PLD participates in and affects the inflammatory process of CRS. (a) Venn map of intersection genes between PLD target genes and CRS-related genes. (b) PPI network map of 19 intersection genes. (c) Eight core targets in the PPI network. (d) GO functional enrichment analysis. (e) Enrichment analysis of KEGG pathway.

**Additional file 3:Table S1.** Patients’ basic information in clinic.

**Additional file 4.** 1483 CRS-related genes and 100 targets of PLD in the human body.

## Data Availability

The original contributions presented in the study are included in the article and Additional file 1. Further inquiries can be directed to the corresponding authors.

## Abbreviations

ARE: Antioxidant response element
ASC: Apoptosis-associated speck-like protein containing CARD
ATP: Adenosine triphosphate
Caspase-1: Cysteinyl aspartate-specific proteases-1
CCK-8: Cell Counting Kit-8
CRS: Chronic rhinosinusitis
DAPI: 4,'6-Diamidino-2-phenylindole
EthD-I: Ethidium homodimer-I
ELISA: Enzyme-linked immunosorbent assay
GSDMD: Gasdermin D
HE: Hematoxylin & eosin
HNEpCs: Human nasal epithelial cells
HO-1: Heme oxygenase-1
IF: Immunofluorescence
IL-1β: Interleukin-1β
IL-18: Interleukin-18
Keap-l: Kelch-like ECH2 associated protein-1
LDH: Lactate dehydrogenase
LPS: Lipopolysaccharide
NLRP3: Nucleotide binding oligomerization domain-like receptor protein 3
Nrf2: Nuclear factor erythroid-2 related factor 2
OD: Optical density
PI: Propidium iodide
PLD: Platycodon D
ROS: Reactive oxygen species
TBHQ: Tert-butylhydroquinone
TEM: Transmission electron microscopy
WB: Western blotting
