# Supplementary material for: Platycodon D protects human nasal epithelial cells from pyroptosis through the Nrf2/HO-1/ROS signaling cascade in chronic rhinosinusitis
Source: Chin Med. 2024 Mar 4;19:40. doi: 10.1186/s13020-024-00897-y (PMC10910709; doi:10.1186/s13020-024-00897-y)
Supplement: Supplementary file 1 — Additional file 1. The specific inclusion criteria and exclusion criteria of patients and the method of network pharmacology. [file 13020_2024_897_MOESM1_ESM.doc]

**Supplementary materials**

**1. Patients information**

1. **Patients with chronic rhinosinusitis with nasal polyps (CRSwNP)**

**Inclusion criteria**

1) Aged 18-65 years old;

2) Patients who meet the diagnostic criteria of EPOS2020 for CRSwNP, plan to undergo surgical treatment, and agree to participate in the study;

3) Patients without consciousness, language, writing, computing, and other obstacles can understand and sign the informed consent.

**Exclusion criteria**

1) Patients combined with allergic rhinitis, fungal rhinitis or nasal malignancy;

2) Patients combined with severe cardiovascular disease or respiratory disease (such as asthma), severe liver or kidney insufficiency, systemic malignancy, etc;

3) Patients with a history of mental disorders;

4) Lactating and pregnant women.

1. **Patients with simple nasal septum deviation**

**Inclusion criteria**

1) Aged 18-65, volunteer to participate in the study;

2) Patients who meet the diagnostic criteria for simple nasal septum deviation, plan to undergo surgical treatment, and agree to join the study;

3) Patients without consciousness, language, writing, computing and other obstacles can understand and sign the informed consent.

**Exclusion criteria**

1) Patients with a history of upper respiratory tract infection in the last three months;

2) Patients combined with CRS (with or without nasal polyps), allergic rhinitis, fungal rhinitis, or nasal malignancy;

3) Patients combined with severe cardiovascular disease or respiratory disease (such as asthma), severe liver or kidney insufficiency, systemic malignancy, and etc.;

4) Patients with a history of mental disorders;

5) Lactating and pregnant women.

**2. Network pharmacology**

**2.1 Prediction of the target of PLD**

Using the Pubchem (https://pubchem.ncbi.nlm.nih.gov/) retrieval of PLD Canonical SMILES serial number, the target of PLD in the human body was predicted via the Swiss Target Prediction database.

**2.2 Acquisition of targets for CRS**

The targets related to "Chronic rhinosinusitis" were searched in GeneCard (https://www.genecards.org/), and the targets of PLD were obtained. Subsequently, the obtained PLD targets were intersected with the CRS-related targets, and a Venn diagram was created using R language. The resulting targets may be the potential targets of PLD in the treatment of CRS.

**2.3 Construction of protein-protein interaction (PPI) network and screening of core target genes**

The intersection genes obtained were imported into the STRING database (https://cn.string-db.org/), with the species set as Homo sapiens. The minimum required interaction score was set to a medium confidence level of 0.400, and unconnected nodes in the network were hidden to generate a protein interaction network of the related targets of PLD in the treatment of CRS. The resulting network was imported into Cytoscape 3.7.1 software, and the MCODE plug-in was used to obtain the core targets of this protein interaction network.

**2.4 Enrichment analysis of core target genes**

Relevant targets were uploaded to the DAVID database (https://david.ncifcrf.gov/), with the species limited to "Homo sapiens.” The official gene symbol was used to amend the name of target genes, and a significance threshold of P < 0.05 was set for the functional enrichment analysis of Gene Ontology (GO) and pathway enrichment analysis of Kyoto Encyclopedia of Genes and Genomes (KEGG). The results were visualized using the R language software (version 4.2.1).

**3. Supplementary Fig.1 information**

**S Fig.1 Network pharmacology confirmed that PLD participates in and affects the inflammatory process of CRS.** (a) Venn map of intersection genes between PLD target genes and CRS-related genes. (b) PPI network map of 19 intersection genes. (c) Eight core targets in the PPI network. (d) GO functional enrichment analysis. (e) Enrichment analysis of KEGG pathway.
