# Supplementary figures and images for: Platycodon D protects human nasal epithelial cells from pyroptosis through the Nrf2/HO-1/ROS signaling cascade in chronic rhinosinusitis
Source: Chin Med. 2024 Mar 4;19:40. doi: 10.1186/s13020-024-00897-y (PMC10910709; doi:10.1186/s13020-024-00897-y)

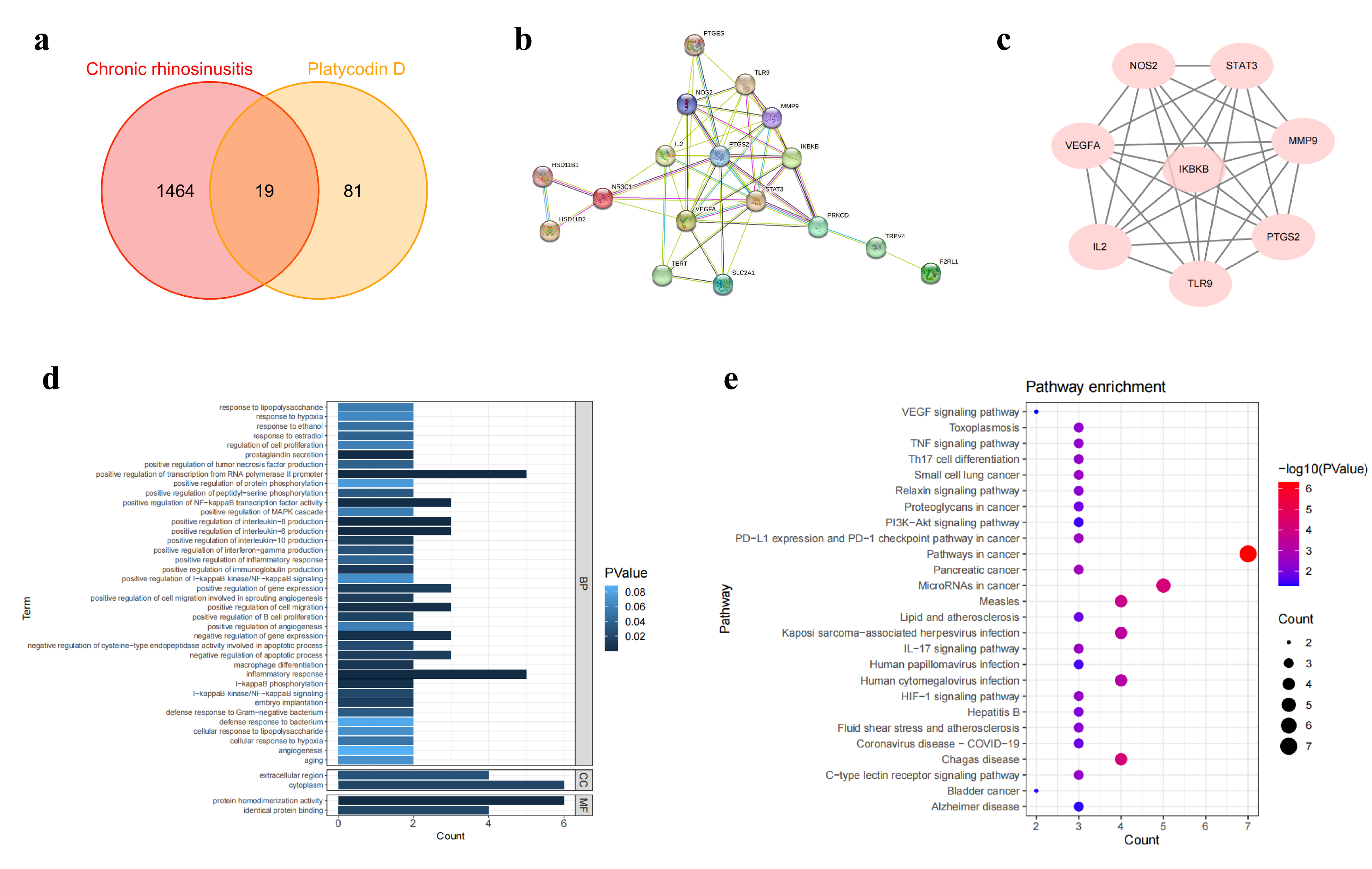

Supplement: Supplementary file 2 — Additional file 2: Fig S1. Network pharmacology confirmed that PLD participates in and affects the inflammatory process of CRS. (a) Venn map of intersection genes between PLD target genes and CRS-related genes. (b) PPI network map of 19 intersection genes. (c) Eight core targets in the PPI network. (d) GO functional enrichment analysis. (e) Enrichment analysis of KEGG pathway. [file 13020_2024_897_MOESM2_ESM.tif]
