# Supplementary material for: Platycodon D protects human nasal epithelial cells from pyroptosis through the Nrf2/HO-1/ROS signaling cascade in chronic rhinosinusitis
Source: Chin Med. 2024 Mar 4;19:40. doi: 10.1186/s13020-024-00897-y (PMC10910709; doi:10.1186/s13020-024-00897-y)
Supplement: Supplementary file 3 — Additional file 3:Table S1. Patients’ basic information in clinic. [file 13020_2024_897_MOESM3_ESM.doc]

**S Table.1 Patients’ basic information in clinic**.

|  | Control group (n = 9) | CRSwNP group (n = 21) | *P value* |
| --- | --- | --- | --- |
| Age(years) |  | | |
| Mean±SD | 42.000 ± 11.640 | 45.476 ± 12.356 | 0.479 |
| Gender |  | | |
| Male | 6 | 13 | / |
| Female | 3 | 8 | / |
| Height(cm) | 168.222 ± 6.180 | 165.905 ± 6.693 | 0.756 |
| Weight(kg) | 76.022 ± 9.274 | 65.762 ± 14.307 | 0.094 |
| Blood cell counts |  | | |
| White blood cell counts  (×10^9^/L, mean±SD) | 6.352 ± 1.020 | 7.260 ± 1.610 | 0.131 |
| Eosinophilic counts  (×10^9^/L, mean±SD) | 0.154 ± 0.101 | 0.277 ± 0.256 | 0.349 |
| Percentage of eosinophils | 2.278% ± 1.534% | 3.700% ± 3.179% | 0.304 |
| Neutrophil count  (×10^9^/L, mean±SD) | 3.574 ± 0.669 | 4.243 ± 1.349 | 0.081 |
| Percentage of neutrophils | 56.211% ± 4.594% | 58.157% ± 11.985% | 0.526 |
